# Ultra-fast, high spatial resolution single-pulse scintillation imaging of synchrocyclotron pencil beam scanning proton delivery

**DOI:** 10.1088/1361-6560/acb753

**Published:** 2023-02-17

**Authors:** Megan Clark, Xuanfeng Ding, Lewei Zhao, Brian Pogue, David Gladstone, Mahbubur Rahman, Rongxiao Zhang, Petr Bruza

**Affiliations:** 1 Dartmouth College, NH, Lebanon; 2 Beaumont Proton Therapy Center, Detroit, MI, United States of America; 3 University of Wisconsin-Madison, Madison, WI, United States of America; 4 Dartmouth Cancer Center, NH, Lebanon; 5 UT Southwestern Medical Cent, United States of America

**Keywords:** proton, synchrocyclotron, PBS, FLASH, scintillation, single-pulse imaging, optical imaging

## Abstract

*Objective.* To demonstrates the ability of an ultra-fast imaging system to measure high resolution spatial and temporal beam characteristics of a synchrocyclotron proton pencil beam scanning (PBS) system. *Approach.* An ultra-fast (1 kHz frame rate), intensified CMOS camera was triggered by a scintillation sheet coupled to a remote trigger unit for beam on detection. The camera was calibrated using the linear (*R* ^2^ > 0.9922) dose response of a single spot beam to varying currents. Film taken for the single spot beam was used to produce a scintillation intensity to absolute dose calibration. *Main results*. Spatial alignment was confirmed with the film, where the *x* and *y*-profiles of the single spot cumulative image agreed within 1 mm. A sample brain patient plan was analyzed to demonstrate dose and temporal accuracy for a clinically-relevant plan, through agreement within 1 mm to the planned and delivered spot locations. The cumulative dose agreed with the planned dose with a gamma passing rate of 97.5% (2 mm/3%, 10% dose threshold). *Significance*. This is the first system able to capture single-pulse spatial and temporal information for the unique pulse structure of a synchrocyclotron PBS systems at conventional dose rates, enabled by the ultra-fast sampling frame rate of this camera. This study indicates that, with continued camera development and testing, target applications in clinical and FLASH proton beam characterization and validation are possible.

## Introduction

Proton pencil beam scanning (PBS) has become widely adopted for its conformal dose delivery and normal tissue sparing capabilities (Zhang *et al* 2009; Yoo *et al* 2020). The current standard of care in PBS treatment utilizes intensity modulated proton therapy (IMPT), which varies the current and energy of each spot to paint a precise dose distribution (Kooy *et al* 2015). Though many reports suggest the dosimetric advantages of IMPT over intensity modulated radiation therapy (IMRT), limitations due to spot size, delivery efficiency, particle range uncertainty, and interplay with internal motion still exist (Ding *et al* 2018, Moreno *et al* 2019). Emerging technologies, such as proton arc therapy, where the proton spot and energy layers are optimized through arc trajectories, and ultra-high dose rate radiotherapy (FLASH-RT), aim to improve the delivery robustness, efficiency, and therapeutic ratio, thus becoming of high interest (Carabe-Fernandez *et al* 2015, Diffenderfer *et al* 2020, Schreuder *et al* 2020, Cunningham *et al* 2021).

While advanced delivery technologies for PBS, such as FLASH-RT and proton arc, have demonstrated appealing potentials to further increase the therapeutic ratio, the existing dosimetry and quality assurance (QA) technologies have struggled to meet new challenges, particularly in the spatial and temporal domain (Ashraf *et al* 2020). FLASH-RT has been demonstrated with high energy proton beams with and without downstream range and intensity modulations. The understanding of the mechanisms behind the FLASH effect when delivered with PBS beams is extremely limited, in part due to the difficulty of characterizing the beam dynamics at the FLASH timescale; therefore, most current studies only do this with simulation (Jolly *et al* 2020). Similarly, further clinical studies are needed to demonstrate the benefits of proton arc to IMPT plans, where current beam stability and pause uncertainties need to be resolved (Carabe-Fernandez *et al* 2015). Developing QA devices and workflows will be essential for the translation of either technique to routine clinical use (Li 2019).

The dynamics of PBS necessitate comprehensive QA of the dosimetry and delivery processes, which are routinely measured with devices based on a range of technologies. Existing dosimetry methods for PBS include high sampling rate ion chambers (IC), parallel-plate strip-segmented IC arrays, scintillator-based sensors, Faraday cups, and simulations (Newhauser *et al* 2015, Han *et al* 2021, IBA Dosimetry, Yang *et al* 2022). The spatiotemporal dynamics introduced by PBS are complicated by the spot position, profile, and dose that is delivered with a unique pulse structure. Current synchrocyclotron-based proton therapy systems, such as the IBA’s ProteusONE superconducting synchrocyclotron accelerator S2C2 used in this study, deliver a high-intensity pulsed beam to each spot in layers via several bursts, with a pulse rep rate and length of 1 kHz and 7 us, respectively (Jolly *et al* 2020, Zhao *et al* 2022). The unique temporal pulse structure of a synchrocyclotron produced proton beam, interplayed with delivery via PBS, introduces complicated spatiotemporal dosimetric characteristics, requiring dosimeters with equally high spatial and temporal resolution. To address the unmet need, this study develops an advanced technique to quantify the full field dose delivery at the kHz delivery rate in PBS systems.

Recently, Yang *et al* published a 2D strip ionization chamber array with high spatiotemporal resolution for PBS imaging (Jolly *et al* 2020). As with all ionization chambers, calibration to dose rate is required and may limit the relevant range of usability. Rahman *et al* demonstrated the utility of a scintillation imaging system with high spatial-temporal resolution for accurate dosimetry of a cyclotron beam (Rahman *et al* 2020). Similarly, Liu *et al* evaluated the effect of the scintillation screen used in a 2D scintillation imaging system on the dose conformity; however, no temporal beam characteristics were evaluated (Liu *et al* 2022). In contrast, Kanouta *et al* report a scintillation-based detector with sub-millisecond temporal resolution, but no ability to get 2D dose distributions (Kanouta *et al* 2022). Most recently, Goddu *et al* published on a scintillation imaging system of a passive scattering proton beam, where the system presented required light tight housing to image and imaged at 264 frames per second (Goddu *et al* 2022). Apart from this research, there have been limited advances in novel FLASH dosimetry methods. As more studies are conducted to evaluate the FLASH effect with protons, accurate and precise dosimetry tools will be required.

This study utilizes similar concepts to the camera technology presented by Rahman *et al*, where a clinical cyclotron beam was imaged at 100 frames per second to validate the proposed monitoring system (Rahman *et al* 2020). The camera implementation presented here substantially differs from the earlier technology, as it is designed towards real-time ultra-high dose rate beam validation of various proton and electron beams. These applications drove the system specifications to achieve over 10 kHz repetition rate and <1 us inter-frame dead time, enabled by a unique intensifier and data processing unit. In this study, we used a 1 kHz framerate to match the output pulse rate of the synchrocyclotron. The camera was designed to allow future image-based real-time interlock functionality, imposing extreme requirements on the data transfer rate (in this case exceeding 16 Gb s^−1^) and transfer fidelity, ensuring no frame packets are lost or require re-translation. Frame rates exceeding 1 kHz, as is enabled with this system, will be required for continuous wave beam applications.

Here we present a first-of-its-kind study that images a pulsed synchrocyclotron beam at a 1 kHz frame rate while capturing 1920 × 1080 pixel images with 1 × 1 mm^2^ spatial resolution. The fast-imaging technique measured dose and spot locations was verified with radiochromic film, treatment planning system generated dose profiles, and irradiator generated log files. The camera also demonstrated dose rate and output per-pulse dynamics imaging at single pulses of the synchrocyclotron PBS system with a brain patient plan.

## Methods and materials

### Experimental setup

**Figure 1.**
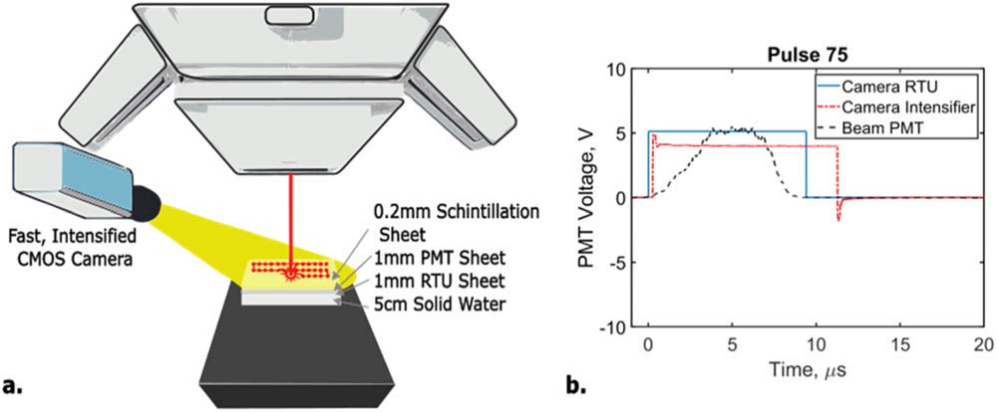
(a) Experimental camera setup of camera facing a scintillation sheet at isocenter. (b) Examples of single pulse response of camera components. ‘Beam PMT’ represents true beam-on time captured with an independent photomultiplier tube (PMT), while ‘Camera RTU’ represents a digitized signal used to trigger the camera intensifier. ‘Camera Intensifier’ signal shows a pulse with pre-defined width that controls the intensifier gate.

The camera system comprises of a high speed CMOS image sensor coupled with a Gen3 intensifier and a lens with focal length of 150 mm (BeamSite Ultra, DoseOptics LLC). 4-lane CXP-12 data backend is used to transfer data to a PC workstation equipped with CXP framegrabber. The camera was implemented in the bunker of a ProteusONE S2C2 synchrocyclotron system at Beaumont Proton Therapy Center in Royal Oak, MI, USA (figure 1(a)). The camera imaged a 0.2 mm thick scintillation sheet (Blue 800, PJ Xray, New Jersey, USA), approximately two meters away from the camera at isocenter. The camera was setup to simulate a clinically relevant orientation, out of the way of patients or practitioners while still enabling capture of high-resolution, minimally distorted images of the scintillation sheet. By being farther away, the neutron damage to the camera system is also reduced. However, there is no fundamental limitation to optimize the positioning of the camera closer fixing it to the gantry, ceiling, and/or wall, with the lens and imaging angle properly adjusted. The current set up was primarily used for considerations on flexibility and proof of concept. To minimize the impact of background illumination in the open imaging system, the camera intensifier was externally triggered to match the proton beam pulses using a remote trigger unit (RTU, DoseOptics LLC) that digitized the temproal beam structure. The RTU utilized two silicon photomultipliers coupled to a 1 mm thick, 200 **×** 250 mm^2^ BC-212 plastic sintillator sheet, optically shielded with opaque white foil (Ashraf *et al* 2022). The camera collected image data at a rate of 1000 frames-per-second (fps), with 1 **×** 1 mm^2^ full-width half maximum (FWHM) spatial resolution and 1920 **×** 1200 pixel count. Temporal beam pulse verification was achieved with a photomultiplier tube (PMT, Hamamatsu), coupled to a separate, identical scintillator sheet as the RTU (figure 1(b)).

Capturing image data at this frame rate, with high spatial resolution is extremely technically challenging. This system only captures data while the beam is on, as demonstrated by figure 1(b) which shows the camera intesifier following the signal from the beam-on signal given from the RTU, to increase the signal to noise ratio. This method enables this unique system to provide temporal and spatial resolution at unprecedented levels, extending the applications of the camera and highlighting the novelty of this setup.

The camera collected images for single spot and scanning beam configurations, and the cumulative images were calibrated and compared to the surface dose calculations from the RayStation treatment planning system (TPS, RaySearch Labratories Stolkholm, Sweeden) and to IBA’s dosimetry LynxPT 2023 (IBA Dosimetry, Herndon, VA) quality assurance device. Temporal agreement was validated through frame-by-frame comparison to the beam spot positions recorded in the IBA system log files and to the planned TPS spot positions. Log files were processed with the open-source software OpenREGGUI and spatially alligned to the quired images in MATLAB (R2019b).

### Imaging processing

**Figure 2.**
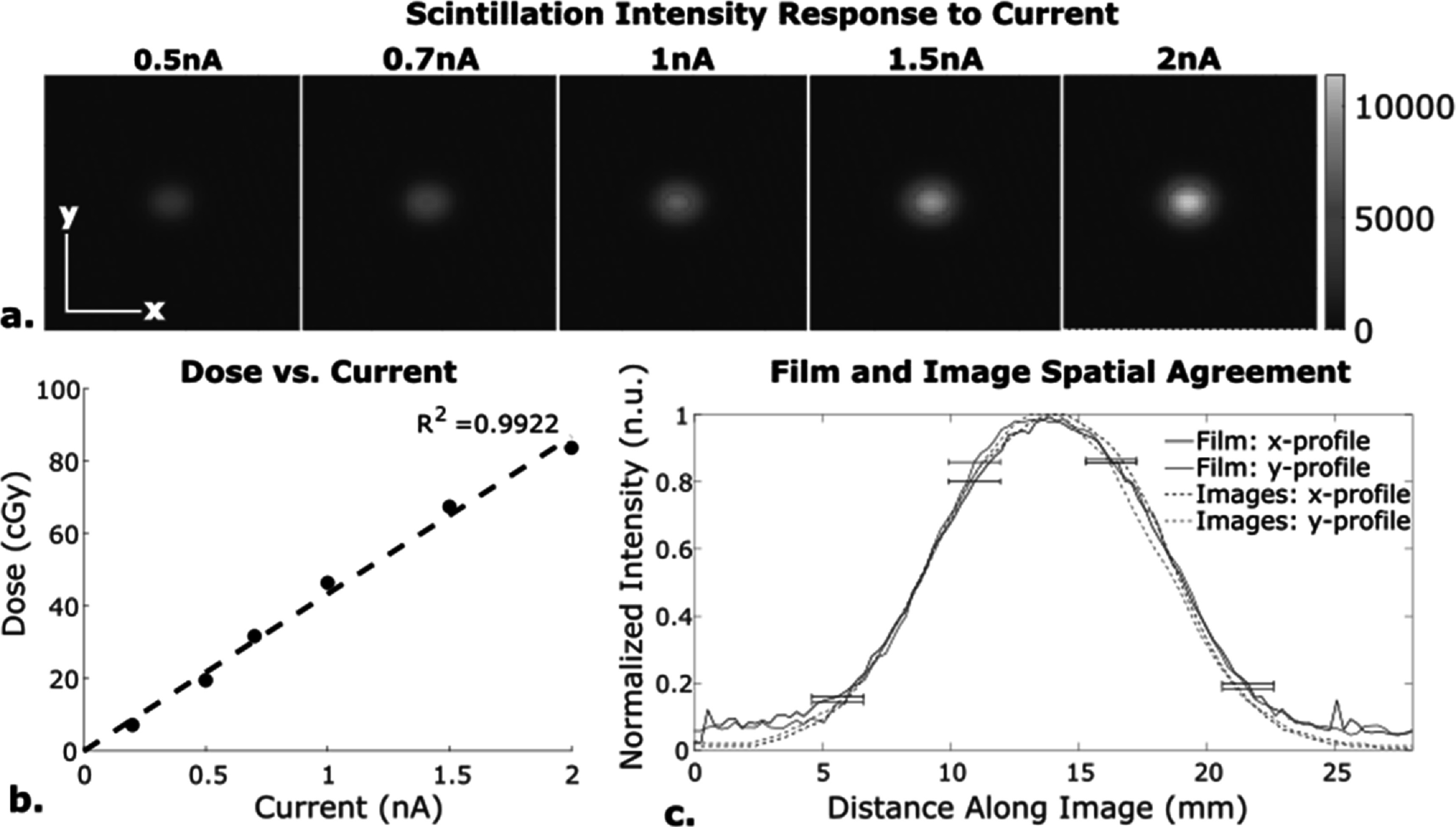
Camera calibration from single-spot beam. (a) Visualization that image intensity, and therefore dose, increased linearly with beam current. (b) Linearity of dose measurements from calibrated images as a function of beam current. (c) *x*–*y* profile agreement between single-spot film and scintillation images.

All image processing was performed in MATLAB (R2019b). Image stacks were offset-corrected with background subtration, spatially transformed with an affine transform, and divided by the flat-field image for spatial and camera nonlinearity correction. The affine transform was used to account for the oblique angle of the camera relative to the couch and to account for observed variations from the flat field, as further described in section 2.3. Pixel-size was correlated to distance using the known spatial resolution of the camera and was verified using film, as shown in figure 2(c). With the image correctly aligned to the true beam geometry, the known dose from the film was related to scintillation intensity, as described in section 2.4.

### Flat-field and spatial calibration

**Figure 3.**
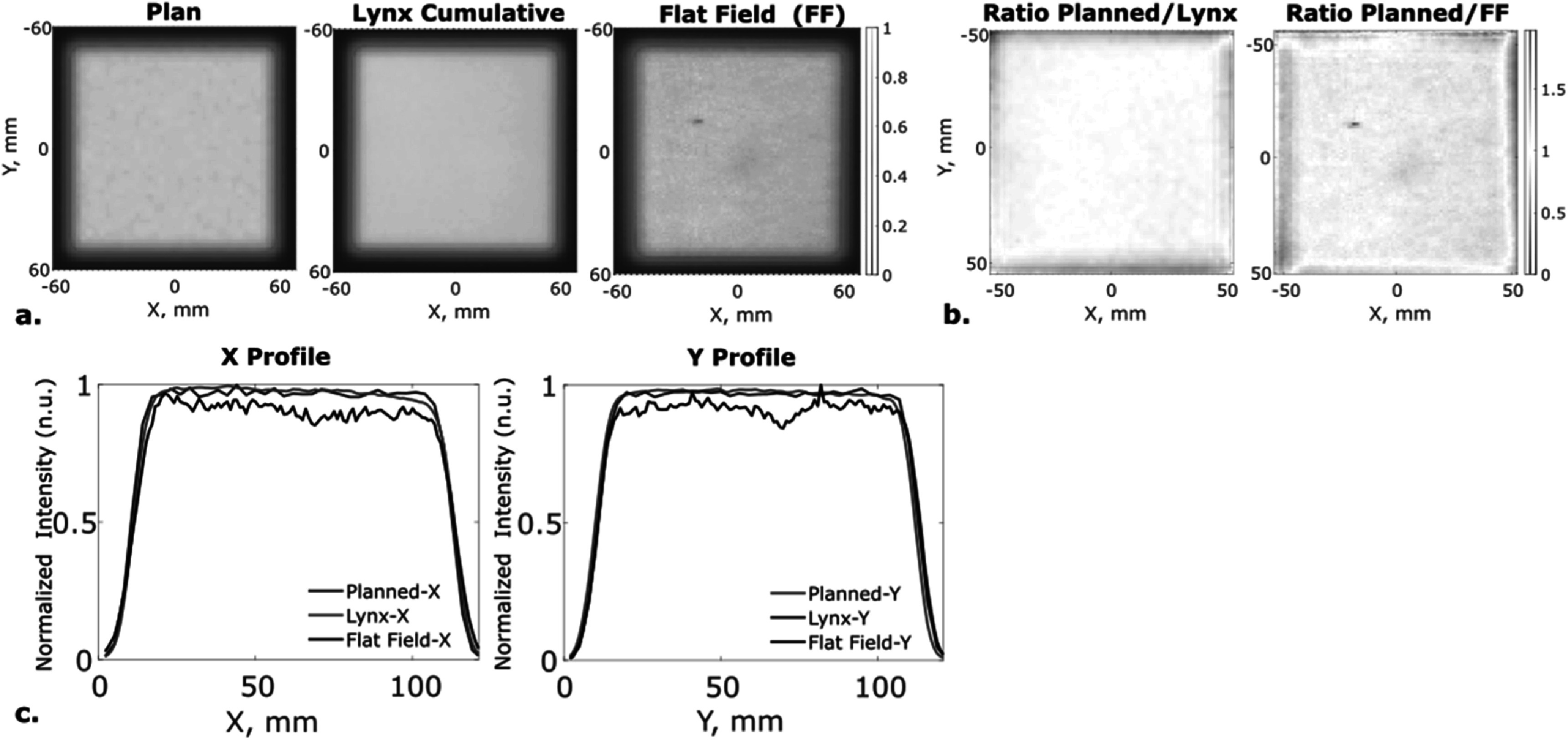
10 × 10 cm^2^ (flat) field spatio-temporal analysis. (a) Normalized planned surface dose, dose delivery recorded with IBA Lynx, and cumulative scintillation, flat field image. (b) Ratio of plan to delivered (Lynx recording) beam and ratio of plan to cumulative image, highlighting increased deviation with scintillation imaging and warranting use as flat field correction. (c) Cross-sectional *X* and *Y* profile recordings of planned, Lynx data, and imaged data.

**Figure 4.**
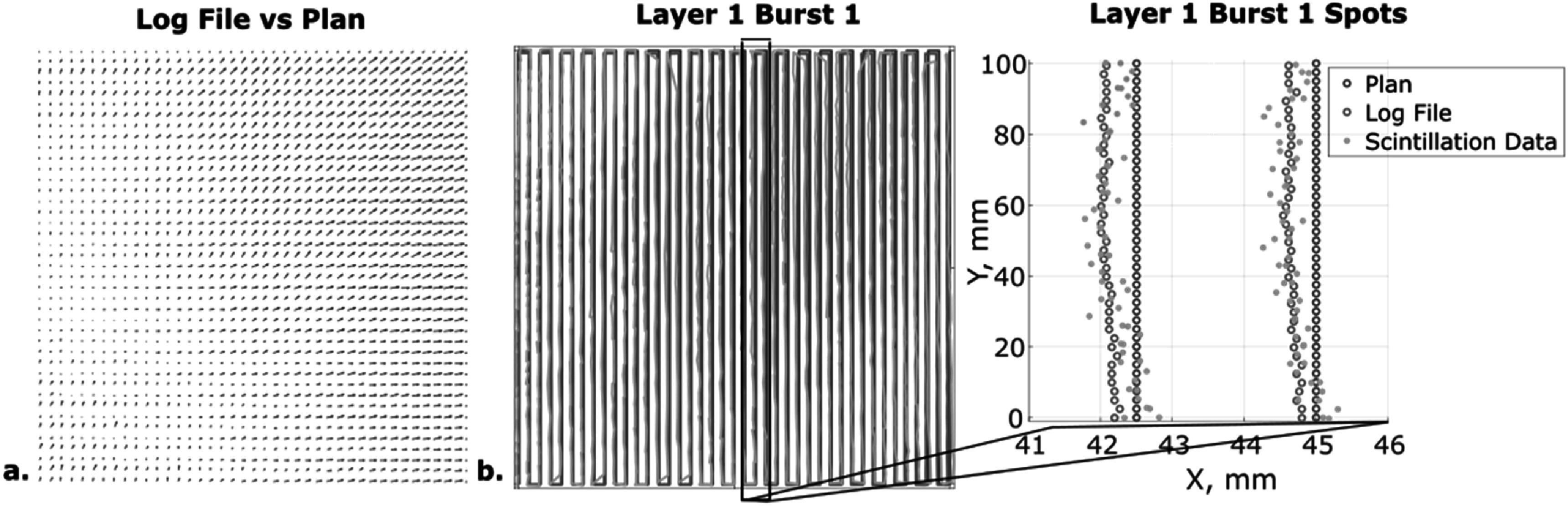
(a) Vector map comparing log file recordings to planned spot positions. (b) Planned versus log file versus imaged scanned beam position and spot-by-spot comparison for the first burst of the energy layer. Image data was processed using a Gaussian-fitting algorithm to localize spot position frame-by-frame.

A 10 × 10 cm^2^ (flat) field was delivered at 227.7 MeV, 2.5 mm spot spacing, and 0.059 MU/spot and imaged at 1000 fps. The cumulative image of this large-field delivery exibited inhomogeneities not seen by the IBA Lynx cumulative delivery measurement file (figure 3). To account for the spatially imhomogeneous dose respose, the 10 × 10 cm^2^ image was used as flat field correction factor when performing dose and dose rate analysis on other imaged deliveries. The 10 × 10 cm^2^ field was also used to optimize the affine transformation, via an internally-developed Gaussian fitting algorithm, to localize the center of the beam spot in each image frame (figure 4). The affine transform was developed by creating a function that tried to match the planned spot positions with the imaged spot positions. By applying this transform to other imaged deliveries, the spatial sensitivity map inherent to the imaging system would be corrected for and not impact the spatial dose distribution.This allowed for accurate frame-by-frame analysis of spot scanning locations, where the center of the individual spots was compared to planned and log file recordings of spot position for temporal accuracy analysis.

### Dose calibration

The scintillation intensity was calibrated to the absolute dose recorded with EBT3 Film. Similar to the method described by Rahman *et al* (2020), where here ${k}$ is the calibration factor, $\sum {D}_{\mathrm{film}}\left(x,y\right)$ is the total dose reported from the optical density of the film at each (*x*, *y*) position, and $\sum {\mathrm{SC}}_{\mathrm{ref}}(x,y)$ is the sum of the scintilltion intensity values at each pixel location (*x*, *y*) of the cumulative single spot image:

\begin{eqnarray*}k=\frac{\displaystyle \sum {D}_{\mathrm{film}}\left(x,y\right)}{\displaystyle \sum {\mathrm{SC}}_{\mathrm{ref}}(x,y)}.\end{eqnarray*}

This calibration factor was multiplied to each pixel in the cumulative image in order to convert the intensity values into dose, measured in cGy. The film was also used to demonstrate strong spatial alignment of the images with the beam. With a camera’s point spread function FWHM of 1 **×** 1 mm^2^, the agreement between the single spot beam and image of the single spot were expected to be within 1 mm (figure 2(c)). The choice of EBT3 Film for reference dosimetry is based on Khachonkhlam *et al*’s conclusion that EBT3 film’s response is independent of radiation type outside the vicinity of the Bragg peak (Khachonkham *et al* 2018). Because we are monitoring surface dose with this camera system, we can consider the characteristic response of EBT3 film as the same as that for electrons. Jaccard *et al* demonstrate agreement in dose to the TPS of film compared to a TLD, concluding that EBT3 film can be used for reference dosimetry in pulsed electron beams, even up to high dose rates (Jaccard *et al* 2017). Surface dosimetry allows analysis of relative beam intensity and positioning in time. While this does not give an absolute measurement of the beam at different depths during the beam delivery time, it does give a robust way to characterize how the delivered beam differed from the planned beam both in space and time.

Dose rate calculations are an advantage of this imaging system, enabled by its high spatial-temporal accuracy. Two different methods to calculate dose rate are included in this analysis to highlight the depth of information this imaging system can provide. First, maximum dose rate is defined as the maximum dose value delivered in one frame, or time interval, at each pixel location divided by the time it took to acquire that individual frame. Due to the difficulties in extracting this information from existing clinical QA devices, a max dose rate map is included as an example. To highlight the accuracy of these calculations, however, a comparison of mean dose rate of different energy layers to the same value calculated with the Lynx and log file data from the clinical devices is included. Mean dose rates are defined as the accumulated dose in a given energy level divided by the total time of delivery for all bursts in that that energy layer (Zhao *et al* 2022).

### Camera response characterization

The expected characteristic camera response was validated with single spot beams at various beam currents. For this calibration, 100 frames were collected at 1 kfps to image single spots delivered at 227.7 MeV, and image intensity/scintillation intensity were expected to be linear with current (Beddar *et al* 1992, Almurayshid *et al* 2017). By applying the intensity-to-dose calibration factor to the cumulative images of the 100 frames, the linearity between dose and current could also be validated. The single spots were also used for spatial profile verification of a single spot, where film taken for the dose calibration was compared to the spatially corrected images.

### Camera performance validation

A brain tumor plan consisting of 14 layers at proton beam energy range of 95–118 MeV was delivered and recorded to validate the camera’s absolute dose response and spot localization accuracy in a clinically relevant scenario. The recorded image stack, consisting of 4200 frames, was spatially-corrected, flat-field normalized, and calibrated to absolute dose as described above. Frame-by-frame analysis was performed to demonstrate the temporal accuracy of the system. For alignment with the TPS, log files, and Lynx, the known resolution (0.5 mm/pixel each) of the DICOM files were used and isocenter was given by the dicom.

## Results

### Intensity and dose linearity

Image intensity and calculated dose were confirmed to be linear with respect to current with *R* ^2^ values of 0.9822 and 0.9922 respectively (figures 2(a)–(b)). The intensity of the center of the beam spot only varried by an average standard deviation of 1.98 counts, indicating strong stability for camera and beam. Spatial agreement was demonstrated with the film and image *x* and *y* spatial profiles of the single spot beam having a mean agreement within 1 mm (figure 2(c)).

### Spatial calibration

The Lynx measurements for a 10 × 10 cm^2^ are included in figure 3 to highlight any discrepancies between the planned and delivered beam. Despite the potential for disagreement, there are clearly spatial inhomogeneities innate to the imaging system which are not present in the 10 × 10 cm^2^ field captured with the Lynx (figures 3(a)–(c)). These imaging system inhomogeneities are used as a correction factor for subsequent imaging acquisitions, as described in section 2.3 Flat field and spatial calibration. Log file comparisons to the plan were also used to analyze recorded differences between planned and delivered beam (figure 4(d)).

To analyze and correct for the disagreement between the plan and log files with the images, the individual spot locations were processed. Planned spot positions agreed with the log file recordings of the spot positions by 0.2 ± 0.2 mm in the *x*-direction and by 0.2 ± 0.1 mm in the *y*-direction, figure 4(a). Similar agreement was found between the planned spot positions and the imaged spot positions, with a deviation of 0.2 ± 0.2 mm in the *x*-direction and 0.4 ± 0.2 mm in the *y*-direction; a deviation between the log files and the images was calculated to be 0.5 ± 0.4 mm deviation in the *x*-direction and 0.6 ± 0.4 mm in the *y*-direction (figure 4(b)). The high agreement found in this frame-by-frame spot localization analysis confirmed the spatio-temporal accuracy of the imaging system.

### Brain patient plan

**Figure 5.**
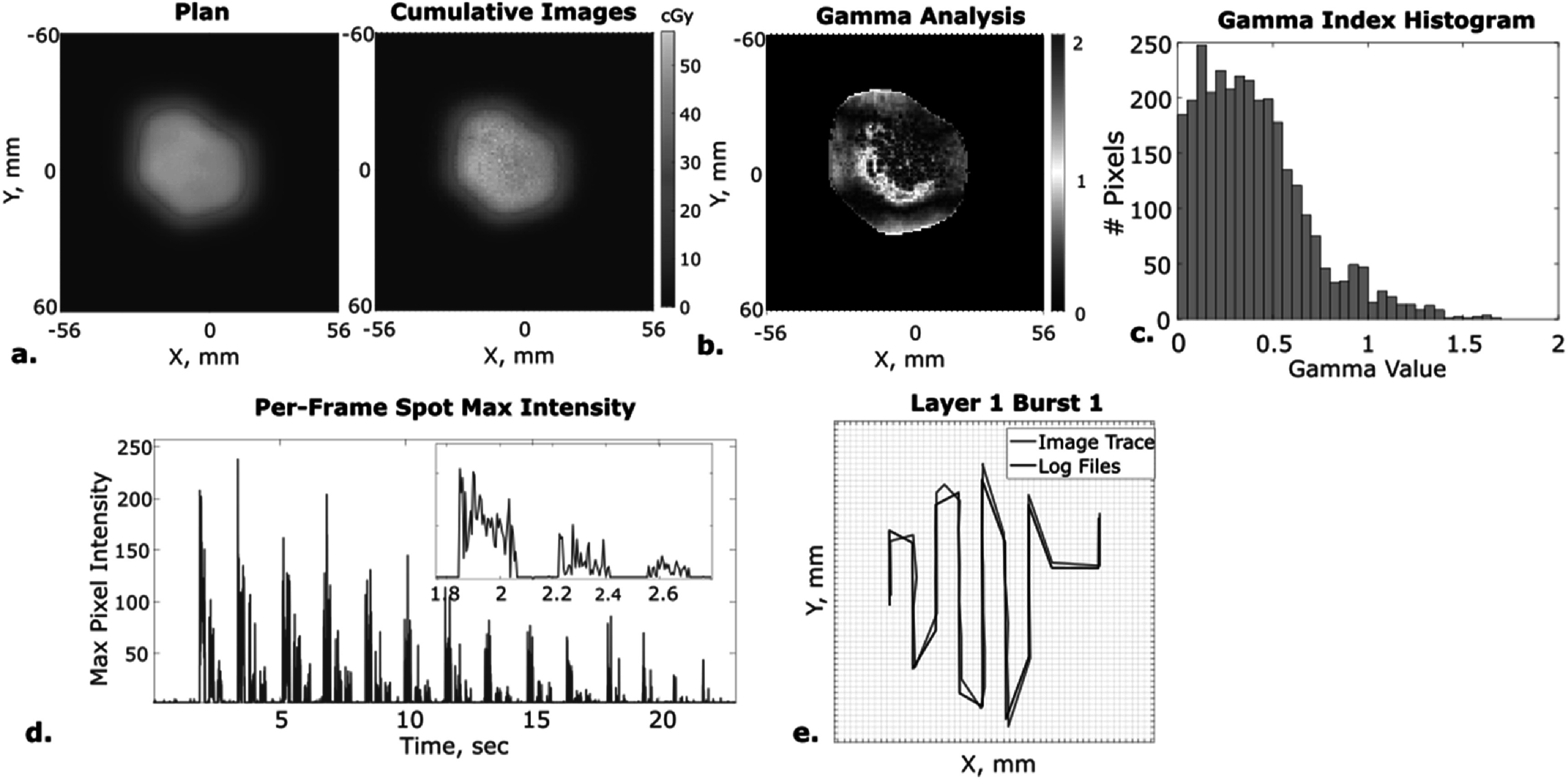
Imaging of first field of clinical brain patient plan delivery. (a) Planned surface dose calculated from TPS and cumulative scintillation image dose calculations. (b) Gamma analysis between plan and cumulative image dose. (c) Gamma index histogram correlating to gamma analysis map. (d) Max scintillation intensity for each image acquired during the entire plan delivery, demonstrating capture of all 14 beam layers. (e) Maximum energy Layer 1, burst 1 beam path imaging comparison to log file recording.

One field (60 cGy) of a clinical brain patient plan was delivered and imaged with this ultra-fast system to demonstrate spatio-temporal dose agreement between the imaging system and the TPS (figure 4). The cumulative dose shows strong agreement within the irradiated field as indicated by a gamma passing rate of 97.5% (with analysis criteria of 2 mm/3% and 10% dose threshold) (figure 5(b)). Because surface dose was being evaluated, a region with small dose distribution gradients, 2D gamma analysis of the global maximum was deemed sufficient.

Each acquired, single-pulse image was flat-field corrected, affine transformed, and compared to the *x*–*y* log file recording of the beam position during delivery, as explained in sections 2.2 and 2.3. The brain plan was delivered in 14 layers, with 3 bursts per layer, each captured with the camera system (figure 5(d)). This imaging system recorded the spot positions used to deliver all layers and bursts, enabling visualization of the different layer’s spatio-temporal structures. Like seen for the 10 × 10 cm^2^ plan, the log file and planned spot positions only deviated by 0.11 ± 0.09 mm in the *x* direction and 0.05 ± 0.03 mm in the *y* direction. The image center-spot positions agreed with the log file recordings by 0.5 ± 0.4 mm in the *x* direction and by 0.5 ± 0.5 in the *y* direction. In all cases, the images were able to locate the beam spot positions within 1 mm.

**Figure 6.**
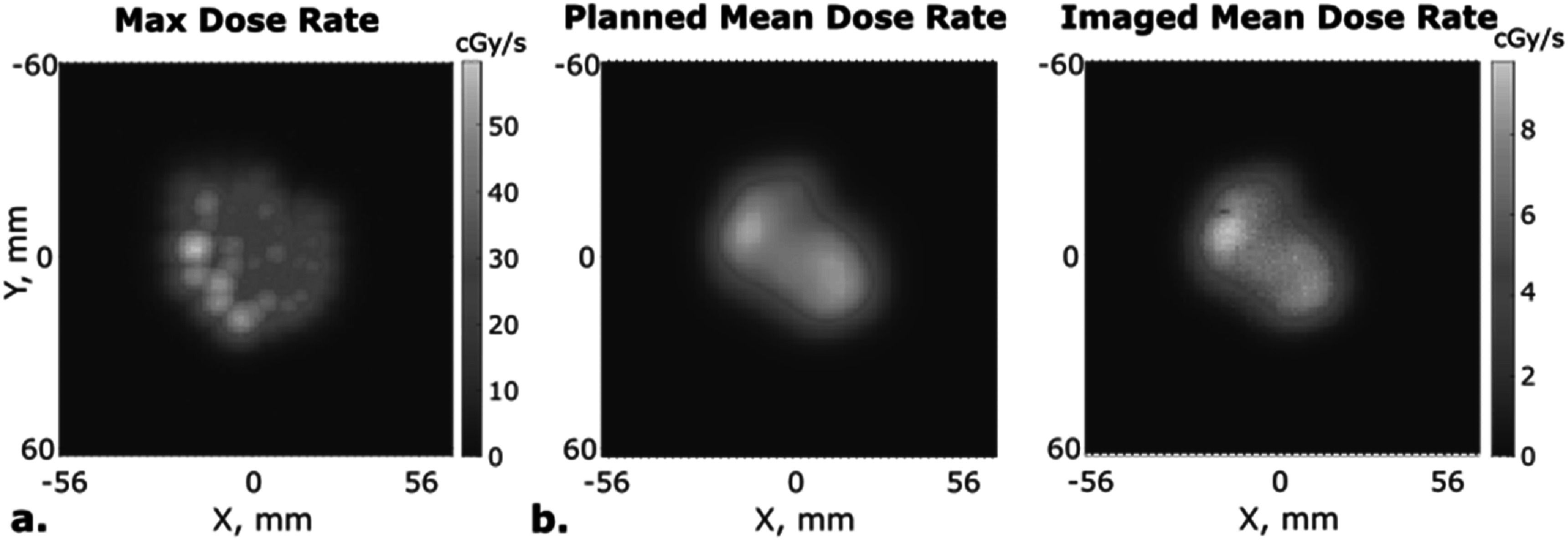
(a) Maximum dose rate calculation from imaging data. (b) Second energy level, mean dose rate calculation from TPS and imaging data.

As discussed in section 2.4, this camera system enables accurate dose rate calculations due to its high spatial and temporal accuracy of dose at the surface. To demonstrate feasability, the maximum dose rate throughout the entire field delivery is shown in figure 6(a). The mean dose rate was calculated for each energy layer, and compared to the energy layer dose calculated by the treatment planning system. As shown in figure 6(b), the maximum value of mean dose rate for all calculated energy levels deviated from the plan by an average of 11%. Potential causes of this deviation are identified in the discussion.

## Discussion

With the acceleration in publications and research work being done in the field of FLASH, dosimetry tools with high spatial and temporal resolution that maintain stability at high dose rates are becoming increasingly import. This work demonstrates the feasability of scintillation imaging as a dosimetry tool that meets these required needs. Sub milimeter resultion in both directions, as higihglighted by the accurate localization of individual spot positions and the beam path and gamma map analysis, emphasize the high spatial resolution of the system. Image acquisition rates of 1 kHz lead to accurate recordings of the temporal beam structure compared to the log files, enabiling dose rate mapping with the ability to accommodate various definitions of dose rate.

In comparison with the existing scintillation dosimetry techniques, this system is able to achieve concurrrent high spatial and temporal resolution. While maintainting these important characteristics, it can act as a passive system, as it could be setup on a tripod or mounted to the wall of a treatment room, out of the way of patients, therapists, and physicians. This application will become important as ultra-high dose rate clinical trials begin and studies continue to be conducted. Existing technology either provides high spatial resolution or high temporal resolution, not both, or requires a unique setup condition that could not accommodate *in vivo* measurements (Jaccard *et al* 2017, Khachonkham *et al* 2018, Goddu *et al* 2022, Kanouta *et al* 2022, Liu *et al* 2022).

While film use is justified in section 2.4, one limitation of this study is the inherent uncertainty in this calibration method. Studies to verify energy dependency of the system and to fully characterize the scintillation intensity-to-dose calibration factor will be important next steps for this imaging system’s development. Similarly, intrinsic error in the log files and planned beam positions from the TPS at the surface, both of which are used for ground-truth comparison, could lead to increased uncertainty and error in our data analysis. Identification and acquisition of better comparison methods, like simulation or other existing dosimetry tools, may enable improved beam characterization going forward; however, these standard methods have the lowest uncertainty given the available QA devices.

Further development of the spot localization algorithm and camera position/orientation correction methods will likely allow this system to develop into an independent dosimetry tool. Similarly, camera calibration and hardware improvements will lead to better per-pulse spot localization and temporal linearity, and testing on a PBS FLASH beam will solidify the use of this system for extended dosimetry applications. Testing system linearity and stability at frame rates exceeding the 1 kHz used here, as the system is capable of, will be another important step in the development and verification of the system’s usable acquisition rate. Advancing this system, with innovative scintillator applications, will also extend the utility of this imaging to *in vivo* measurements.

## Conclusion

This study demonstrated the first spatio-temporal dose distribution measurements of a pulsed, synchrocyclotron beam made with an ultra-fast scintillation imaging system synchronized to the proton pulses. This method accurately measured the surface dose profile and per-pulse spot positions of a clinical patient treatment plan, suggesting utility as an all-in-one PBS quality assurance tool. Additionally, this camera system was able to produce accurate maximum and mean dose rate values, as compared to the TPS. As UHDR therapy become of increasing interest to the radiation therapy community, quality assurace devices that are able to produce this breadth and accuracy of information will be of the utmost importance. Because of the ability of this camera to image the complicated beam structure of a synchrocyclotron, it shows extreme promise for UHDR and proton arc beam analysis.

## Funding

NIH Grants P30CA023108, U01CA260446, R43CA268466.
